# Supplementary material for: Kerr reversal in Josephson meta-material and traveling wave parametric amplification
Source: Nat Commun. 2022 Apr 1;13:1737. doi: 10.1038/s41467-022-29375-5 (PMC8975858; doi:10.1038/s41467-022-29375-5)
Supplement: Supplementary file 1 — Supplementary Information : Kerr reversal in Josephson meta-material and traveling wave parametric amplification [file 41467_2022_29375_MOESM1_ESM.pdf]

Supplementary Information : Kerr reversal in Josephson meta-material and traveling wave parametric amplification

Arpit Ranadive,[1,*] Martina Esposito,[1,2] Luca Planat,[1] Edgar Bonet,[1]
Cécile Naud,[1] Olivier Buisson,[1] Wiebke Guichard,[1] and Nicolas Roch[1]

[1] *Univ. Grenoble Alpes, CNRS, Grenoble INP, Institut Néel, 38000 Grenoble, France*
[2] *CNR-SPIN, c/o Complesso di Monte S. Angelo, via Cinthia - 80126 - Napoli, Italy*

I. EXPERIMENTAL SETUP

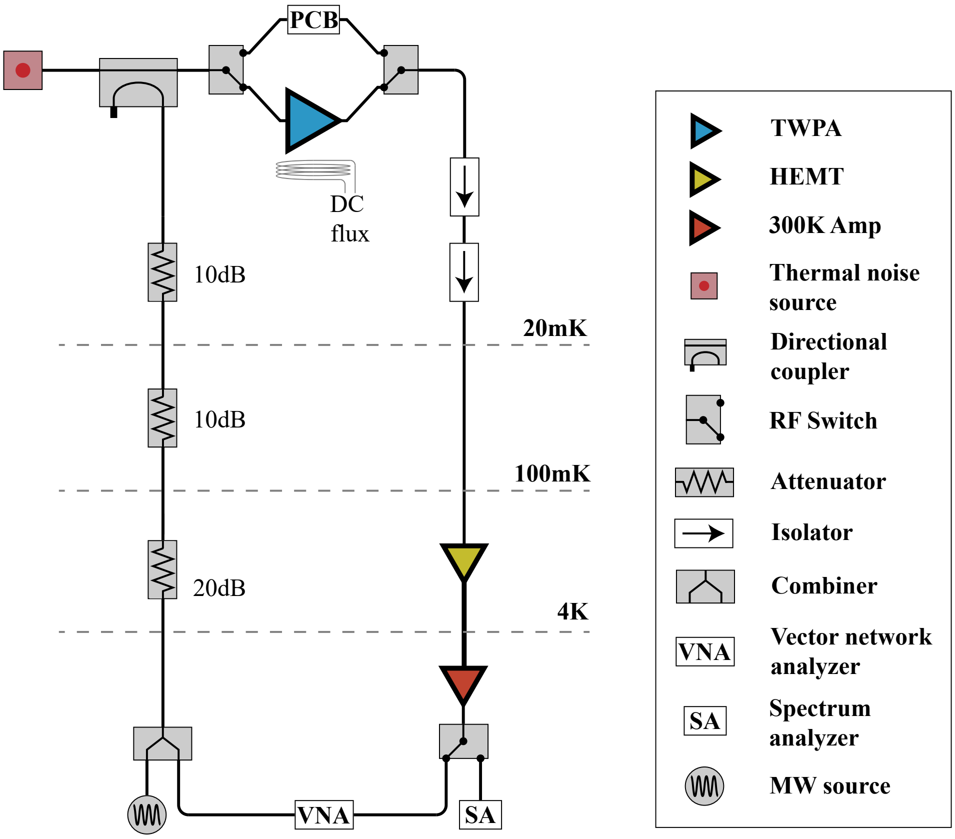

FIG. 1. **Sketch of the experimental setup.**

II. DEVICE CHARACTERIZATION IN LOW POWER REGIME

Device characteristics in linear regime, i.e. with no pumping, are depicted in Fig. 2. The net transmission for the device is obtained by subtracting the transmission of a dummy PCB device measured using exactly the same set-up (see Fig. 1 for detailed schematic). Device transmission versus frequency and flux is shown in Fig. 2(a). Two vertical cuts, corresponding to zero and half quantized flux, are shown in the inset. At half quantized flux, which corresponds to the optimal flux point for reversed Kerr phase matching, we find that losses are roughly 1 dB per GHz . Such values are comparable with previously demonstrated TWPAs based on Josephson meta-materials [1, 2].

We also measure the phase of the signal transmitted through the device, θ_{device}, using a dummy PCB device as reference. The measured phase is directly linked to the dispersion relation, $k(\omega) = \theta_{\text{device}}(\omega)/N$, where N is the number of SNAILs in the transmission line. At a fixed flux, the frequency dependent phase evolution is fitted to the linear dispersion relation,

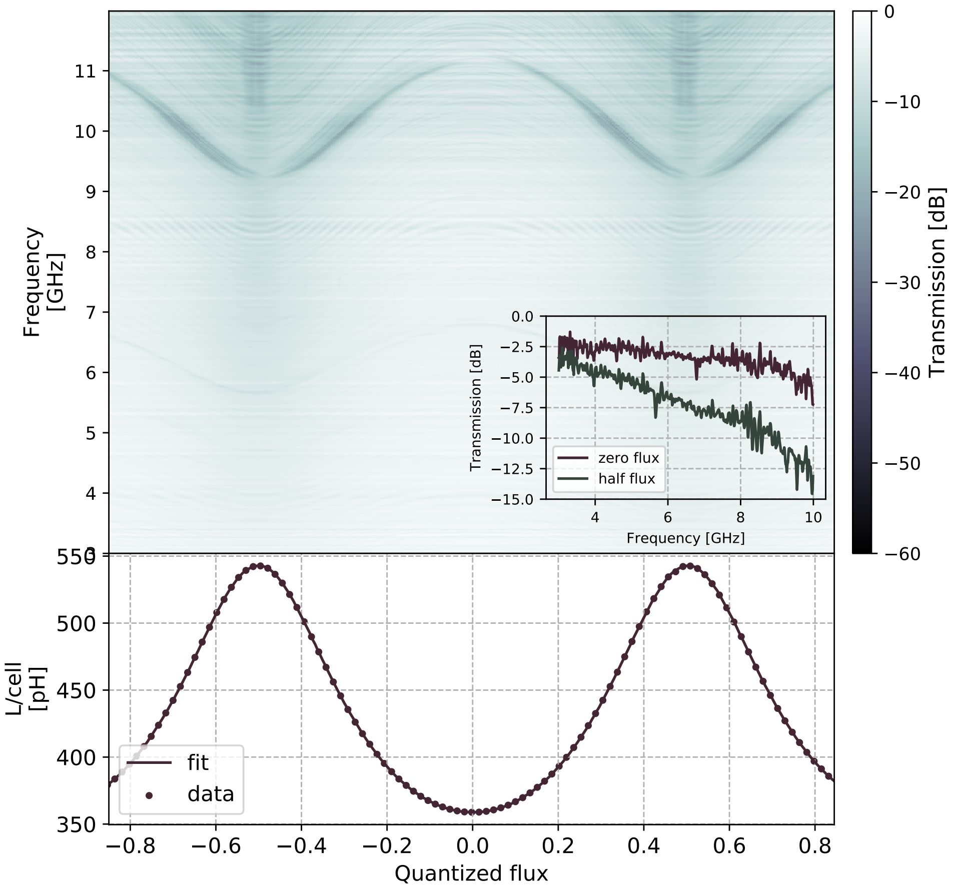

FIG. 2. **Linear characterization.** (a) Calibrated transmission through the device as function of external magnetic flux and frequency. (inset) Calibrated transmission through the device for two selected flux values, $\Phi_{\mathrm{ext}}/\Phi_0 = 0$ and $\Phi_{\mathrm{ext}}/\Phi_0 = 0.5$. (b) Inductance L of one unit cell as function of flux, each dot is obtained by fitting the measured linear transmitted phase at a fixed flux with the dispersion relation model. The solid line is the fit of the data (dotted curve) by using the theoretical expression of L as a function of the external magnetic flux.

$$\theta_{\mathrm{device}} = \theta_0 + N \frac{\sqrt{LC_g}\,\omega}{\sqrt{1 - \omega^2/(LC_J)}}\,, \tag{1}$$

using the cell inductance, L, as fit parameter, and fixing the other parameters with the design/fabrication values: $N = 700$, $C_g = 250$ fF and $C_J = 50$ fF. The intercept θ_0 is added to the model to account for the $2n\pi$ uncertainty introduced by phase wrapping.

The values of L obtained for various flux points from the fits are shown in Fig. 2(b) (dotted curve). This data is then fitted using the theoretical expression for L as a function of the external magnetic flux,

$$L(\Phi_{\mathrm{ext}}) = \frac{\Phi_0}{2\pi I_0\,\widetilde{\alpha}(\Phi_{\mathrm{ext}})}\,, \tag{2}$$

with $\widetilde{\alpha}(\Phi_{\mathrm{ext}})$ defined in in the article and using the asymmetry ratio r and the critical current I_0 as fit parameters. From the best fit we obtain $I_0 = 2.19\,\mu$A and $r = 0.07$, comparable with the values expected by design.

We can also calculate the plasma frequency, $\omega_J = 1/\sqrt{LC_J}$, which varies between 30.5 GHz at half flux and

37.5 GHz at zero flux. These high plasma frequencies give nearly flat flux-dependent transmission as shown in Fig. 2(a).

III. SUPPRESSION OF SECOND ORDER NONLINEARITY

SNAILs exhibit both, second and third order nonlinearities, which are responsible for three wave and four wave mixing processes, respectively. The meta-material presented in the article constitutes in SNAILs with alternating flux polarity, as depicted in the main text. This results in neighboring cells experiencing opposite induced flux reaction. As second order nonlinearity is an odd function of flux, this leads to adjacent cells exhibiting second order nonlinearity with opposite sign; and its effective suppression over meta-material [3]. Second harmonic generation, three wave mixing process involving conversion of two probe photon into one at twice the probe frequency, is proportional to the second order nonlinearity of the medium [4]. Fig 3 shows simulated comparison of output between meta-material with and without alternating flux polarity when a -90dBm probe tone is applied at 4 GHz at the input. As expected, absence of significant second harmonic generation is observed when second order nonlinearity is suppressed. The simulation was computed using transient analysis in wrspice tool [5, 6] for meta-material with 700 SNAILs and Josephson junction parameters similar to the presented device.

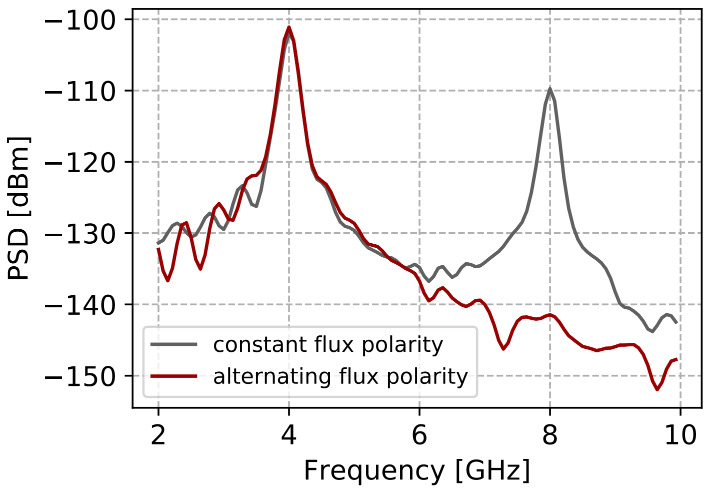

FIG. 3. Simulated comparison between second harmonic generation in meta-materials with and without flux polarity inversion.

IV. GAIN RIPPLES AND CHARACTERISTIC IMPEDANCE OF TWPAS

Primary origin of gain ripples in TWPAs is improper impedance matching in the devices [7]. Fig. 4 (a) depicts simulated characteristic impedance of a transmission line with and without modulation for dispersion engineering. The modulation was applied with a 10 percent amplitude on inductances and capacitances in telegrapher model of transmission line with cell inductance of 240 pH and device length equivalent to presented TWPA. Fig. 4 (b) depicts the gap in the transmission opened by this modulation.

Simulations were performed using standard ABCD matrix modeling of the transmission line [8]. Fig. 4 (c) depicts peak to peak gain ripple as a function of impedance mismatch between environment and TWPA with 20 dB forward gain. These values were obtained by comparing the constructive and destructive interference between forward propagating wave (ζ_0) and first order reflection correction (ζ_1) [7],

$$\begin{aligned}\zeta_0 &= g(1-\Gamma_1)(1-\Gamma_2)A_{in},\\ \zeta_1 &= g^2\Gamma_1\Gamma_2(1-\Gamma_1)(1-\Gamma_2)A_{in},\end{aligned} \tag{3}$$

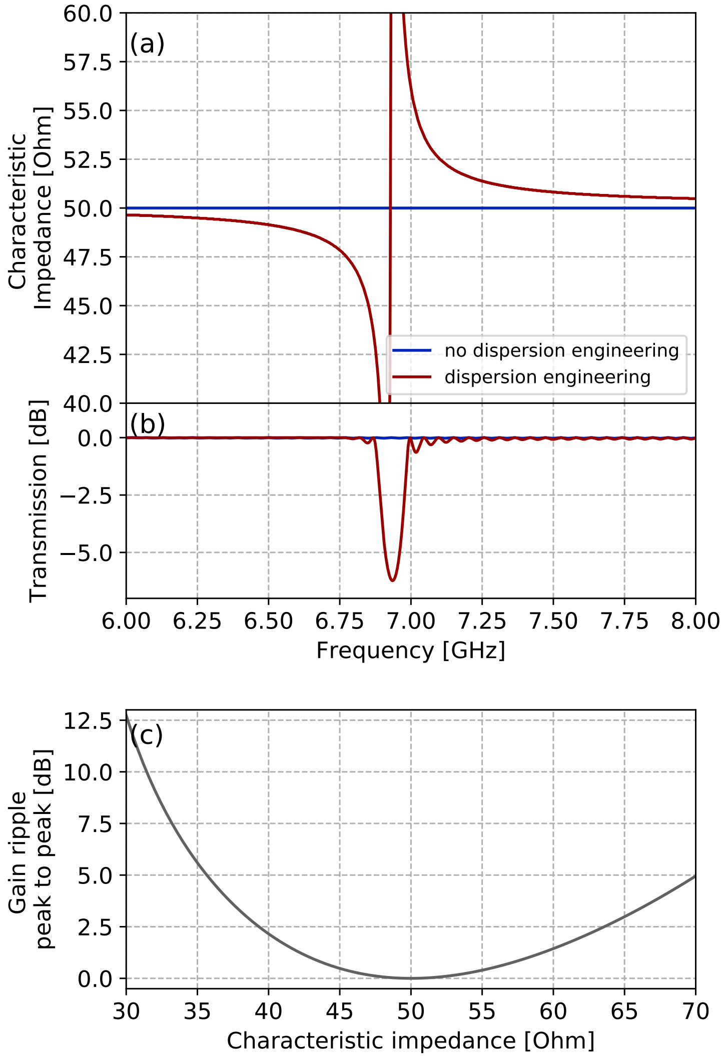

FIG. 4. **Characteristic impedance.** (a) Simulated characteristic impedance of a transmission line with and without dispersion engineering. (b) Gap in the transmission created by modulation. (c) Simulated peak to peak gain ripples versus characteristic impedance of the TWPA with 20 dB gain. Gain ripples are minimized when the TWPA impedance matches 50 Ω environment impedance.

where g is the linear amplitude gain, A_{in} is the input amplitude, Γ_1 and Γ_2 are reflection coefficients at input and output interface respectively.

Due to nearly flat impedance profile, the reversed Kerr amplifier has better impedance matching to the environment, and hence, should exhibit lower reflections and gain ripples compared to TWPAs with dispersion engineering in identical environment. The measured improvement in gain ripples is shown in the main text, obtained by measuring the two devices, dispersion engineered TWPA and reversed Kerr TWPA in identical packaging.

V. SIMULATED GAIN

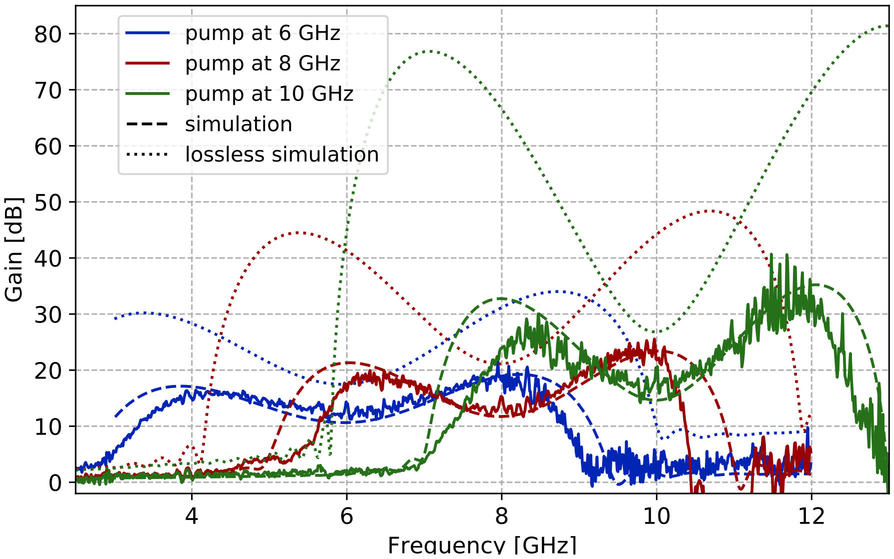

FIG. 5. **Gain simulation.** Comparison between gain simulation using losseless model, dotted curves, and complete theory, dashed curves. Experimental data are indicated with continuous curves. It is necessary to include the transmission losses of the device into the gain modeling for accurate quantitative simulation of the gain.

---

* Corresponding author: arpit.ranadive@neel.cnrs.fr

[1] C. Macklin, K. O'Brien, D. Hover, M. E. Schwartz, V. Bolkhovsky, X. Zhang, W. D. Oliver, and I. Siddiqi, "A near – quantum-limited Josephson traveling-wave parametric amplifier," Science, vol. 350, p. 307, 2015.

[2] L. Planat, E. Al-Tavil, J. P. Martínez, R. Dassonneville, F. Foroughi, S. Léger, K. Bharadwaj, J. Delaforce, V. Milchakov, C. Naud, O. Buisson, W. Hasch-Guichard, and N. Roch, "Fabrication and characterization of aluminum squid transmission lines," Phys. Rev. Applied, vol. 12, p. 064017, Dec 2019.

[3] A. B. Zorin, "Quasi-phasematching in a poled traveling-wave Josephson parametric amplifier with three-wave mixing," arXiv:2101.11697 [cond-mat, physics:physics], Jan. 2021. arXiv: 2101.11697.

[4] J. A. Armstrong, N. Blgemeergen, J. Ducuing, and P. S. Pershan, "Interactions between Light Waves in a Nonlinear Dielectric*," Physical Review, vol. 127, no. 6, 1962.

[5] S. Whiteley, "Josephson junctions in SPICE3," IEEE Trans. Magn., vol. 27, pp. 2902–2905, Mar. 1991.

[6] T. Dixon, J. W. Dunstan, G. B. Long, J. M. Williams, P. J. Meeson, and C. D. Shelly, "Capturing Complex Behaviour in Josephson Travelling Wave Parametric Amplifiers," arXiv.org, Dec. 2019.

[7] L. Planat, Resonant and traveling-wave parametric amplification near the quantum limit. PhD thesis, UGA Grenoble, 2020.

[8] D. M. Pozar, Microwave Engineering. John Wiley & Sons, Inc., fourth edi ed., 2010.
